# 2-[2-(Methyl­sulfan­yl)benzimidazol-1-yl]ethanol

**DOI:** 10.1107/S1600536810001960

**Published:** 2010-01-23

**Authors:** Ludovic Akonan, Kouassi Yves Guillaume Molou, Adjo Adohi-Krou, Akoun Abou, Abodou Jules Tenon

**Affiliations:** a Laboratoire de Cristallographie et Physique Moléculaire, UFR SSMT, Université de Cocody 22 BP 582 Abidjan 22, Côte d’Ivoire; b Laboratoire de Chimie Organique, UFR SSMT, Université de Cocody 22 BP 582 Abidjan 22, Côte d’Ivoire

## Abstract

In the title compound, C_10_H_12_N_2_OS, the asymmetric unit consists of two independent mol­ecules. In the crystal structure, mol­ecules form *R*_4_^4^(28) centrosymmetric tetra­mers *via* O—H⋯N hydrogen bonds. These tetra­mers are stacked along the *c* axis *via* C—H⋯O hydrogen bonds. C—H⋯π and π–π inter­actions are also present; in the latter, the centroid–centroid distances are 4.075 (1) and 3.719 (1) Å.

## Related literature

For the biological activity of compounds having benzimidazole ring systems, and a related structure, see: Akkurt *et al.* (2006 ▶). For other studies of the biological activity of benzimidazoles, see: Küçükbay *et al.* (2003 ▶), (2004 ▶); Puratchikody *et al.* (2008 ▶). For hydrogen-bond graph sets, see: Bernstein *et al.* (1995 ▶).
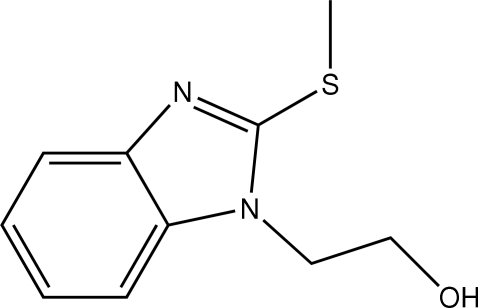


## Experimental

### 

#### Crystal data

C_10_H_12_N_2_OS
*M* *_r_* = 208.28
Triclinic, 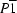

*a* = 9.3235 (2) Å
*b* = 9.7659 (2) Å
*c* = 11.4588 (3) Å
α = 78.0849 (9)°
β = 88.9066 (8)°
γ = 88.1399 (9)°
*V* = 1020.25 (4) Å^3^
*Z* = 4
Mo *K*α radiation
μ = 0.29 mm^−1^
*T* = 223 K
0.20 × 0.20 × 0.15 mm

#### Data collection

Nonius KappaCCD diffractometer
13769 measured reflections
5257 independent reflections
3996 reflections with *I* > 2σ(*I*)
*R*_int_ = 0.036

#### Refinement

*R*[*F* ^2^ > 2σ(*F* ^2^)] = 0.041
*wR*(*F* ^2^) = 0.102
*S* = 0.96
5242 reflections
261 parameters
H atoms treated by a mixture of independent and constrained refinement
Δρ_max_ = 0.51 e Å^−3^
Δρ_min_ = −0.32 e Å^−3^

### 

Data collection: *COLLECT* (Nonius, 2001 ▶); cell refinement: *DENZO*/*SCALEPACK* (Otwinowski & Minor, 1997 ▶); data reduction: *DENZO*/*SCALEPACK*; program(s) used to solve structure: *SIR92* (Altomare *et al.*, 1994 ▶); program(s) used to refine structure: *CRYSTALS* (Betteridge *et al.*, 2003 ▶); molecular graphics: *ORTEP-3 for Windows* (Farrugia, 1997 ▶) and *PLATON* (Spek, 2009 ▶); software used to prepare material for publication: *CRYSTALS*.

## Supplementary Material

Crystal structure: contains datablocks global, I. DOI: 10.1107/S1600536810001960/wn2372sup1.cif

Structure factors: contains datablocks I. DOI: 10.1107/S1600536810001960/wn2372Isup2.hkl

Additional supplementary materials:  crystallographic information; 3D view; checkCIF report

## Figures and Tables

**Table 1:** Hydrogen-bond geometry (Å, °) *Cg*1 and *Cg*2 are the centroids of the N1*A*-C3*A*-N2*A*-C6*A*-C5*A* and C5*A*—C10*A* rings, respectively.

*D*—H⋯*A*	*D*—H	H⋯*A*	*D*⋯*A*	*D*—H⋯*A*
O1*B*—H1*B*⋯N2*A*^i^	0.95 (3)	1.88 (3)	2.825 (3)	174 (3)
O1*A*—H1*A*⋯N2*B*	1.01 (3)	1.80 (3)	2.808 (3)	175 (3)
C4*A*—H41*A*⋯O1*A*^ii^	0.95	2.42	3.366 (3)	174
C4*A*—H43*A*⋯*Cg*2^iii^	0.95	2.86	3.627 (2)	139
C4*B*—H43*B*⋯*Cg*1	0.95	2.86	3.486 (2)	125
C10*B*—H10*B*⋯*Cg*2^iv^	0.95	2.74	3.631 (2)	157

Symmetry codes: (i) 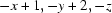; (ii) 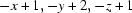; (iii) 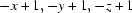; (iv) 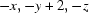.

**Fig. 1.**
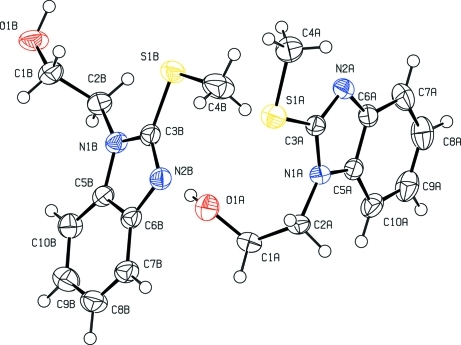
The structure of the asymmetric unit of the title compound, showing the atomic labeling scheme, with displacement ellipsoids drawn at the 50% probability level. H atoms are shown as spheres of arbitrary radius.

**Fig. 2.**
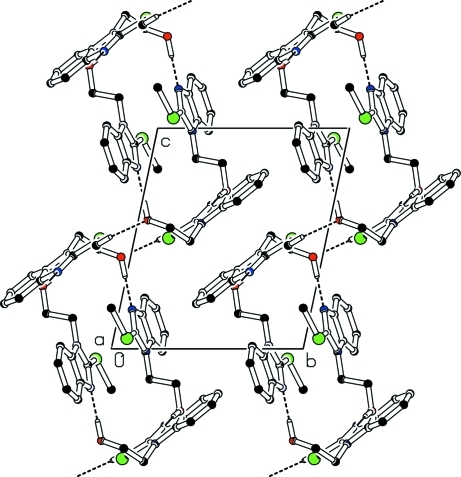
Crystal packing, viewed down the a axis, showing the zigzag infinite chain of cyclic tetramers along the c axis. Dashed lines indicate hydrogen bonds. H atoms not involved in hydrogen bonds have been omitted for clarity.

**Fig. 3.**
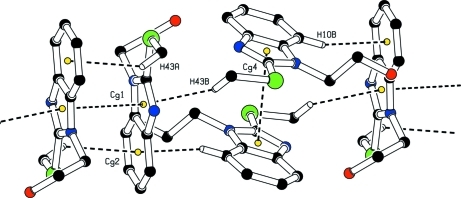
Crystal packing, showing the π–π and C—H···π stacking interactions. The yellow dots are the centroids of benzene and imidazole rings. H atoms not involved in C—H···π interactions have been omitted for clarity.

## supplementary crystallographic information

### Comment

Numerous compounds having benzimidazole ring systems possess versatile pharmacological activities such as antiviral, anthelmintic, spasmolytic, antihypertensive and vasodilator (Akkurt *et al.*, 2006). It has also been reported that many benzimidazole derivatives have antimicrobial and antifungal activities (Küçükbay *et al.*, 2003, 2004, Puratchikody *et al.*, 2008). Therefore, the synthesis of new benzimidazole derivatives is of considerable interest. In order to explore new benzimidazole properties, the title compound has been synthesized and its crystal structure determined.

The two independent molecules in the asymmetric unit of the title compound and the atomic labeling scheme are shown in Fig.1. In this structure, the nine-membered benzimidazole ring systems (N1A/C3A/N2A/C6A/C7A/C8A/C9A/C10A/C5A, N1B/C3B/N2B/C6B/C7B/C8B/C9B/C10B/C5B) of both independent molecules are essentially planar, the maximum deviation from planarity being, respectively, 0.016 (2) Å for atom C8A and 0.078 (16) Å for atom C3B. These two ring systems make a dihedral angle of 73.95 (6)°.

In the crystal structure, we observe the formation of *R*_4_^4^(28) centrosymmetric tetramers (Bernstein *et al.*, 1995) *via* O—H···N hydrogen bonds. The tetramers are linked by two symmetric C—H···O hydrogen bonds to form a zigzag infinite chain along the *c* axis. The supramolecular aggregation is completed by the presence of C—H···π interactions (Table 1) and π–π stacking between two parallel imidazole rings. The centroid···centroid distance of those rings, **Cg*1*···**Cg*1*(1 - *x*,1 - *y*,1 - *z*) and **Cg*4*···**Cg*4*(-*x*,2 - *y*,-*z*) are 4.075 (1) Å and 3.719 (1) Å, respectively  (Fig.3).

### Experimental

2-Chloroethanol (1.6 ml, 24.4 mmol) and potasium carbonate (1.68 g, 12.2 mmol) were added to 2-methylsulfanyl-1*H*-benzimidazole (1 g, 6.1 mmol) in dimethyl sulfoxide (DMSO) (5 ml). The reaction mixture was successively agitated for 30 min at room temperature and at 323 K for 24 h. 50 ml of water was then added to the reaction mixture, and the products were extracted with dichloromethane (3 × 50 ml). The combined organic extracts were washed with brine (10 g of sodium chloride in 100 ml of water), dried (Na_2_SO_4_) and evaporated under reduced pressure. The residue was purified by column chromatography on silica gel (elution: hexane/ethyl acetate (70:30, *v*/*v*)) and the title compound resulted as a brown powder (0.77 g, 61%) with a melting point of 409 K. The brown powder was dissolved in ethanol/hexane (3:1, *v*/*v*) and, after four days, brown crystals suitable for single-crystal X-ray diffraction analysis were obtained.

### Refinement

The H atoms bonded to O1A and O1B were located in a difference Fourier map; their positional parameters and *U*_iso_ were refined freely. Other H atoms were placed at calculated positions, with C—H = 0.95 Å and refined using a riding model, with *U*_iso_(H) constrained to be 1.2*U*_eq_(C).

### Crystal data

**Table:**

C_10_H_12_N_2_OS	*Z* = 4
*M**_r_* = 208.28	*F*(000) = 440
Triclinic, *P*1	*D*_x_ = 1.356 Mg m^−^^3^
Hall symbol: -P 1	Melting point: 409 K
*a* = 9.3235 (2) Å	Mo *K*α radiation, λ = 0.71073 Å
*b* = 9.7659 (2) Å	Cell parameters from 13769 reflections
*c* = 11.4588 (3) Å	θ = 2–29°
α = 78.0849 (9)°	µ = 0.28 mm^−^^1^
β = 88.9066 (8)°	*T* = 223 K
γ = 88.1399 (9)°	Prism, brown
*V* = 1020.25 (4) Å^3^	0.20 × 0.20 × 0.15 mm

### Data collection

**Table:**

Nonius KappaCCD diffractometer	3996 reflections with *I* > 2σ(*I*)
Radiation source: fine-focus sealed tube	*R*_int_ = 0.036
graphite	θ_max_ = 29.1°, θ_min_ = 1.8°
φ and ω scans	*h* = −12→12
13769 measured reflections	*k* = −12→12
5257 independent reflections	*l* = −15→15

### Refinement

**Table:**

Refinement on *F*^2^	Primary atom site location: structure-invariant direct methods
Least-squares matrix: full	Secondary atom site location: difference Fourier map
*R*[*F*^2^ > 2σ(*F*^2^)] = 0.041	Hydrogen site location: inferred from neighbouring sites
*wR*(*F*^2^) = 0.102	H atoms treated by a mixture of independent and constrained refinement
*S* = 0.96	Method = Modified Sheldrick *w* = 1/[σ^2^(*F*^2^) + (0.04*P*)^2^ + 0.62*P*], where *P* = [max(*F*_o_^2^,0) + 2*F*_c_^2^]/3
5242 reflections	(Δ/σ)_max_ = 0.001
261 parameters	Δρ_max_ = 0.51 e Å^−^^3^
0 restraints	Δρ_min_ = −0.32 e Å^−^^3^
88 constraints	

### Special details

**Table:**

Experimental. ^1^H NMR (DMSO-d_6_, 300 MHz, p.p.m.) δ: 2.71 (s, 3H, CH_3_); 3.68–3.74 (m 2H, CH_2_O, J_CH2—CH2_ = 5.7 Hz and J_CH2—OH_ = 5.4 Hz); 4.17 (t, 2H, CH_2_N, J_CH2—CH2_ = 5.7 Hz); 5.00 (t, 1H, OH, J_CH2—OH_ = 5.4 Hz); 7.13–7.17 and 7.46–7.55 (m, 4H, C_6_H_4_). ^13^C NMR (DMSO-d_6_, 300 MHz, p.p.m.) δ: 14.35 (CH_3_); 46.25 (CH_2_N); 59.14 (CH2O); 109.75, 117.31, 121.14, 121.21, 136.75, 142.92 (C_6_H_5_); 152.48 (C═N).
Refinement. The 15 reflections 1 0 0; -1 1 0; 0 1 0; 1 1 0; -1 - 1 1; 0 - 1 1; 1 - 1 1; -1 0 1; 0 0 1; 1 0 1; -1 1 1; 0 1 1; 1 1 1; 0 0 2; 0 1 2 have been measured with too low intensities. It might be caused by some systematical error, probably by shielding by a beam stop of these diffractions. They were not used in the refinement.

### Fractional atomic coordinates and isotropic or equivalent isotropic displacement parameters (Å^2^)

**Table:**

	*x*	*y*	*z*	*U*_iso_*/*U*_eq_	
S1A	0.49131 (5)	0.82212 (5)	0.49825 (4)	0.0390	
C3A	0.42886 (16)	0.69994 (15)	0.42198 (14)	0.0286	
N1A	0.28691 (13)	0.66663 (13)	0.43140 (12)	0.0286	
C5A	0.26838 (17)	0.57367 (15)	0.35708 (15)	0.0298	
C6A	0.40349 (17)	0.55494 (15)	0.30706 (15)	0.0309	
N2A	0.50366 (14)	0.63619 (13)	0.34868 (12)	0.0309	
C7A	0.4221 (2)	0.46382 (18)	0.22858 (18)	0.0430	
C8A	0.3031 (2)	0.39422 (19)	0.20412 (19)	0.0507	
C9A	0.1686 (2)	0.41504 (19)	0.25355 (19)	0.0481	
C10A	0.14723 (19)	0.50586 (17)	0.33090 (17)	0.0386	
C2A	0.17826 (17)	0.70834 (17)	0.51181 (15)	0.0336	
C1A	0.07853 (17)	0.82574 (17)	0.45194 (16)	0.0354	
O1A	0.15279 (14)	0.95034 (13)	0.41601 (12)	0.0405	
C4A	0.67938 (19)	0.8087 (2)	0.4688 (2)	0.0463	
S1B	0.34070 (5)	1.07040 (5)	0.04701 (4)	0.0402	
C3B	0.16057 (17)	1.10450 (15)	0.07442 (14)	0.0299	
N2B	0.09036 (15)	1.06622 (14)	0.17658 (12)	0.0325	
C6B	−0.04765 (17)	1.12427 (16)	0.15371 (14)	0.0307	
C5B	−0.05673 (17)	1.19801 (16)	0.03565 (14)	0.0306	
N1B	0.07856 (14)	1.18307 (13)	−0.01412 (12)	0.0313	
C2B	0.12112 (19)	1.23474 (17)	−0.13814 (14)	0.0357	
C1B	0.1892 (2)	1.37598 (19)	−0.15947 (16)	0.0419	
O1B	0.21179 (14)	1.42576 (15)	−0.28301 (12)	0.0521	
C10B	−0.18151 (19)	1.26805 (18)	−0.01093 (16)	0.0395	
C9B	−0.2986 (2)	1.2612 (2)	0.06571 (18)	0.0458	
C8B	−0.2918 (2)	1.1879 (2)	0.18335 (18)	0.0449	
C7B	−0.16712 (19)	1.11853 (18)	0.22973 (16)	0.0381	
C4B	0.3871 (2)	0.9554 (2)	0.18458 (19)	0.0578	
H1B	0.305 (3)	1.398 (3)	−0.306 (3)	0.091 (9)*	
H1A	0.136 (3)	0.992 (3)	0.329 (3)	0.094 (9)*	
H10A	0.0555	0.5211	0.3642	0.0468*	
H9A	0.0895	0.3657	0.2337	0.0576*	
H8A	0.3134	0.3303	0.1520	0.0612*	
H7A	0.5130	0.4501	0.1934	0.0516*	
H10B	−0.1862	1.3181	−0.0913	0.0468*	
H9B	−0.3860	1.3078	0.0373	0.0552*	
H8B	−0.3748	1.1855	0.2331	0.0540*	
H7B	−0.1630	1.0688	0.3102	0.0456*	
H41A	0.7284	0.8715	0.5062	0.0552*	
H42A	0.6966	0.8319	0.3851	0.0552*	
H43A	0.7132	0.7157	0.4993	0.0552*	
H41B	0.4855	0.9276	0.1822	0.0696*	
H42B	0.3707	1.0025	0.2486	0.0681*	
H43B	0.3296	0.8751	0.1961	0.0681*	
H11A	0.0039	0.8396	0.5063	0.0420*	
H12A	0.0383	0.8011	0.3838	0.0420*	
H21A	0.2257	0.7382	0.5744	0.0408*	
H22A	0.1226	0.6293	0.5442	0.0408*	
H21B	0.0383	1.2421	−0.1863	0.0432*	
H22B	0.1881	1.1693	−0.1609	0.0432*	
H11B	0.1277	1.4401	−0.1289	0.0504*	
H12B	0.2787	1.3675	−0.1200	0.0504*	

### Atomic displacement parameters (Å^2^)

**Table:**

	*U*^11^	*U*^22^	*U*^33^	*U*^12^	*U*^13^	*U*^23^
S1A	0.0358 (2)	0.0417 (2)	0.0440 (3)	−0.00593 (17)	−0.00121 (18)	−0.01874 (19)
C3A	0.0264 (7)	0.0263 (7)	0.0320 (8)	−0.0010 (5)	−0.0012 (6)	−0.0030 (6)
N1A	0.0250 (6)	0.0282 (6)	0.0324 (7)	−0.0025 (5)	0.0027 (5)	−0.0059 (5)
C5A	0.0301 (8)	0.0237 (7)	0.0345 (8)	−0.0019 (6)	−0.0017 (6)	−0.0030 (6)
C6A	0.0304 (8)	0.0254 (7)	0.0370 (9)	0.0010 (6)	−0.0024 (6)	−0.0067 (6)
N2A	0.0258 (6)	0.0304 (7)	0.0371 (7)	0.0003 (5)	0.0009 (5)	−0.0085 (5)
C7A	0.0469 (10)	0.0357 (9)	0.0496 (11)	0.0062 (7)	−0.0007 (8)	−0.0174 (8)
C8A	0.0676 (14)	0.0342 (9)	0.0557 (12)	0.0016 (9)	−0.0119 (10)	−0.0213 (8)
C9A	0.0518 (11)	0.0344 (9)	0.0596 (12)	−0.0108 (8)	−0.0156 (10)	−0.0107 (8)
C10A	0.0331 (8)	0.0319 (8)	0.0487 (10)	−0.0086 (6)	−0.0055 (7)	−0.0017 (7)
C2A	0.0312 (8)	0.0368 (8)	0.0311 (8)	−0.0020 (6)	0.0077 (7)	−0.0034 (6)
C1A	0.0288 (8)	0.0419 (9)	0.0362 (9)	0.0013 (6)	0.0049 (7)	−0.0107 (7)
O1A	0.0449 (7)	0.0375 (6)	0.0389 (7)	−0.0012 (5)	−0.0030 (6)	−0.0068 (5)
C4A	0.0330 (9)	0.0471 (10)	0.0608 (13)	−0.0082 (7)	−0.0080 (8)	−0.0140 (9)
S1B	0.0384 (2)	0.0427 (2)	0.0365 (2)	0.00559 (17)	0.00863 (18)	−0.00300 (17)
C3B	0.0355 (8)	0.0259 (7)	0.0286 (8)	−0.0031 (6)	0.0039 (6)	−0.0065 (6)
N2B	0.0381 (7)	0.0304 (7)	0.0284 (7)	−0.0011 (5)	0.0044 (6)	−0.0051 (5)
C6B	0.0365 (8)	0.0270 (7)	0.0296 (8)	−0.0048 (6)	0.0043 (6)	−0.0079 (6)
C5B	0.0341 (8)	0.0292 (7)	0.0292 (8)	−0.0060 (6)	0.0040 (6)	−0.0072 (6)
N1B	0.0349 (7)	0.0315 (7)	0.0263 (7)	−0.0031 (5)	0.0037 (5)	−0.0036 (5)
C2B	0.0414 (9)	0.0400 (9)	0.0245 (8)	−0.0014 (7)	0.0047 (7)	−0.0041 (6)
C1B	0.0409 (10)	0.0404 (9)	0.0397 (10)	−0.0038 (7)	0.0071 (8)	0.0021 (7)
O1B	0.0349 (7)	0.0628 (9)	0.0443 (8)	0.0089 (6)	0.0116 (6)	0.0195 (6)
C10B	0.0398 (9)	0.0407 (9)	0.0367 (9)	−0.0002 (7)	−0.0023 (7)	−0.0052 (7)
C9B	0.0358 (9)	0.0508 (11)	0.0519 (12)	0.0036 (8)	0.0000 (8)	−0.0137 (9)
C8B	0.0390 (10)	0.0491 (10)	0.0491 (11)	−0.0024 (8)	0.0116 (8)	−0.0170 (8)
C7B	0.0433 (10)	0.0378 (9)	0.0336 (9)	−0.0055 (7)	0.0104 (7)	−0.0088 (7)
C4B	0.0442 (11)	0.0745 (14)	0.0448 (12)	0.0157 (10)	0.0037 (9)	0.0072 (10)

### Geometric parameters (Å, °)

**Table:**

S1A—C3A	1.7383 (16)	S1B—C3B	1.7369 (16)
S1A—C4A	1.7851 (19)	S1B—C4B	1.788 (2)
C3A—N1A	1.3700 (19)	C3B—N2B	1.321 (2)
C3A—N2A	1.321 (2)	C3B—N1B	1.368 (2)
N1A—C5A	1.384 (2)	N2B—C6B	1.396 (2)
N1A—C2A	1.461 (2)	C6B—C5B	1.398 (2)
C5A—C6A	1.396 (2)	C6B—C7B	1.396 (2)
C5A—C10A	1.396 (2)	C5B—N1B	1.390 (2)
C6A—N2A	1.397 (2)	C5B—C10B	1.388 (2)
C6A—C7A	1.395 (2)	N1B—C2B	1.458 (2)
C7A—C8A	1.382 (3)	C2B—C1B	1.509 (2)
C7A—H7A	0.950	C2B—H21B	0.950
C8A—C9A	1.392 (3)	C2B—H22B	0.950
C8A—H8A	0.950	C1B—O1B	1.413 (2)
C9A—C10A	1.385 (3)	C1B—H11B	0.950
C9A—H9A	0.950	C1B—H12B	0.950
C10A—H10A	0.950	O1B—H1B	0.95 (3)
C2A—C1A	1.510 (2)	C10B—C9B	1.382 (3)
C2A—H21A	0.950	C10B—H10B	0.950
C2A—H22A	0.950	C9B—C8B	1.391 (3)
C1A—O1A	1.402 (2)	C9B—H9B	0.950
C1A—H11A	0.950	C8B—C7B	1.385 (3)
C1A—H12A	0.950	C8B—H8B	0.950
O1A—H1A	1.01 (3)	C7B—H7B	0.950
C4A—H41A	0.950	C4B—H41B	0.950
C4A—H42A	0.950	C4B—H42B	0.950
C4A—H43A	0.950	C4B—H43B	0.950
			
C3A—S1A—C4A	100.28 (8)	C3B—S1B—C4B	100.22 (9)
S1A—C3A—N1A	119.56 (12)	S1B—C3B—N2B	126.69 (13)
S1A—C3A—N2A	126.71 (12)	S1B—C3B—N1B	119.73 (12)
N1A—C3A—N2A	113.67 (14)	N2B—C3B—N1B	113.53 (14)
C3A—N1A—C5A	106.27 (13)	C3B—N2B—C6B	104.41 (13)
C3A—N1A—C2A	127.66 (14)	N2B—C6B—C5B	110.28 (14)
C5A—N1A—C2A	125.82 (13)	N2B—C6B—C7B	129.65 (15)
N1A—C5A—C6A	105.66 (13)	C5B—C6B—C7B	120.07 (16)
N1A—C5A—C10A	131.55 (15)	C6B—C5B—N1B	105.33 (14)
C6A—C5A—C10A	122.79 (15)	C6B—C5B—C10B	122.56 (15)
C5A—C6A—N2A	110.23 (13)	N1B—C5B—C10B	132.10 (15)
C5A—C6A—C7A	120.05 (15)	C5B—N1B—C3B	106.45 (13)
N2A—C6A—C7A	129.72 (15)	C5B—N1B—C2B	126.32 (14)
C6A—N2A—C3A	104.17 (13)	C3B—N1B—C2B	127.15 (14)
C6A—C7A—C8A	117.44 (17)	N1B—C2B—C1B	113.41 (14)
C6A—C7A—H7A	120.6	N1B—C2B—H21B	108.5
C8A—C7A—H7A	122.0	C1B—C2B—H21B	108.4
C7A—C8A—C9A	121.94 (17)	N1B—C2B—H22B	108.5
C7A—C8A—H8A	119.0	C1B—C2B—H22B	108.5
C9A—C8A—H8A	119.0	H21B—C2B—H22B	109.5
C8A—C9A—C10A	121.66 (17)	C2B—C1B—O1B	109.85 (15)
C8A—C9A—H9A	119.2	C2B—C1B—H11B	109.4
C10A—C9A—H9A	119.2	O1B—C1B—H11B	109.5
C5A—C10A—C9A	116.10 (17)	C2B—C1B—H12B	109.4
C5A—C10A—H10A	122.0	O1B—C1B—H12B	109.3
C9A—C10A—H10A	121.9	H11B—C1B—H12B	109.5
N1A—C2A—C1A	113.63 (13)	C1B—O1B—H1B	110.3 (18)
N1A—C2A—H21A	108.4	C5B—C10B—C9B	116.51 (17)
C1A—C2A—H21A	108.3	C5B—C10B—H10B	121.8
N1A—C2A—H22A	108.5	C9B—C10B—H10B	121.7
C1A—C2A—H22A	108.5	C10B—C9B—C8B	121.79 (18)
H21A—C2A—H22A	109.5	C10B—C9B—H9B	119.1
C2A—C1A—O1A	110.78 (13)	C8B—C9B—H9B	119.1
C2A—C1A—H11A	109.2	C9B—C8B—C7B	121.60 (17)
O1A—C1A—H11A	109.2	C9B—C8B—H8B	119.2
C2A—C1A—H12A	109.1	C7B—C8B—H8B	119.2
O1A—C1A—H12A	109.0	C6B—C7B—C8B	117.47 (17)
H11A—C1A—H12A	109.5	C6B—C7B—H7B	121.3
C1A—O1A—H1A	110.8 (16)	C8B—C7B—H7B	121.3
S1A—C4A—H41A	109.5	S1B—C4B—H41B	109.5
S1A—C4A—H42A	109.5	S1B—C4B—H42B	109.5
H41A—C4A—H42A	109.5	H41B—C4B—H42B	109.5
S1A—C4A—H43A	109.5	S1B—C4B—H43B	109.4
H41A—C4A—H43A	109.5	H41B—C4B—H43B	109.5
H42A—C4A—H43A	109.5	H42B—C4B—H43B	109.5

### Hydrogen-bond geometry (Å, °)

**Table:**

*Cg*1 and *Cg*2 are the centroids of the N1A-C3A-N2A-C6A-C5A and C5A—C10A rings, respectively.

**Table:**

*D*—H···*A*	*D*—H	H···*A*	*D*···*A*	*D*—H···*A*
O1B—H1B···N2A^i^	0.95 (3)	1.88 (3)	2.825 (3)	174 (3)
O1A—H1A···N2B	1.01 (3)	1.80 (3)	2.808 (3)	175 (3)
C4A—H41A···O1A^ii^	0.95	2.42	3.366 (3)	174
C4A—H43A···*Cg*2^iii^	0.95	2.86	3.627 (2)	139
C4B—H43B···*Cg*1	0.95	2.86	3.486 (2)	125
C10B—H10B···*Cg*2^iv^	0.95	2.74	3.631 (2)	157

Symmetry codes: (i) −*x*+1, −*y*+2, −*z*; (ii) −*x*+1, −*y*+2, −*z*+1; (iii) −*x*+1, −*y*+1, −*z*+1;  (iv) −*x*, −*y*+2, −*z*.
